# Risk Factors, Clinical Outcomes, and Medical Costs of Pelvic Infection After Open Pelvic Fractures: A 7-Year Retrospective Observational Study at a Single Trauma Center

**DOI:** 10.3390/healthcare14101328

**Published:** 2026-05-13

**Authors:** Donghwan Choi, Jungsub So, Won Tae Cho, Hyung Keun Song, Kyoungwon Jung

**Affiliations:** Division of Trauma Surgery, Department of Surgery, Ajou University School of Medicine, Suwon 16499, Republic of Korea; claptonc@naver.com (D.C.); wontaecho@aumc.ac.kr (W.T.C.); ostrauma@aumc.ac.kr (H.K.S.); jake98@aumc.ac.kr (K.J.)

**Keywords:** medical cost, open pelvic fractures, pelvic infection, pelvic sepsis, soft tissue infection

## Abstract

**Highlights:**

**What are the main findings?**

- Pelvic infection occurred in 50% of open pelvic fractures and was independently associated with older age, elevated serum lactate, Gustilo–Anderson grade III injury, and anorectal injury.
- Pelvic infection nearly tripled hospital stay, number of surgeries, and total medical costs compared with non-infected cases.

**What are the implications of the main findings?**

- Early identification of high-risk patients may aid prevention strategies, including prompt wound management and optimized antibiotic therapy.
- Preventing pelvic infection in open pelvic fractures may reduce healthcare resource utilization and overall treatment costs.

**Abstract:**

**Background/Objectives:** Patients with unstable pelvic fractures typically die of hemorrhagic shock, whereas those with open pelvic fractures (OPF) more commonly die of pelvic infections (PIs) and pelvic sepsis (PS). We examined the clinical outcomes of PI in patients with OPFs. **Methods:** Patients with OPFs treated at our hospital between March 2016 and February 2023 were retrospectively reviewed. Factors associated with PI were identified using logistic regression analysis. **Results:** A total of 44 patients with OPFs were included, of whom 22 developed PIs. The number of patients with Gustilo–Anderson grade III was higher in the PI group than in the non-PI group (n = 18 vs. 8, *p* = 0.008). Similarly, anorectal injury was more frequent in the PI group than in the non-PI group (n = 15 vs. 3, *p* = 0.001). Multivariate logistic regression identified age (odds ratio 1.082 [95% confidence interval 1.020–1.148], *p* = 0.009), serum lactate level (1.319 [0.992–1.755], *p* = 0.018), presence of Gustilo–Anderson grade III (7.467 [0.987–56.517], *p* = 0.052), and anorectal injury (36.468 [3.107–427.991], *p* = 0.004) as independent risk factors for PI. Hospital length of stay, overall medical costs, and number of surgeries were 2.8 (84.0 vs. 30.5 days, *p* = 0.002), 2.9 (95,812 vs. 33,224 USD, *p* = 0.001), and 2.9 (13.0 vs. 4.5, *p* < 0.001) times higher in the PI group than in the non-PI group, respectively. **Conclusions:** Age and anorectal injury were significantly associated with pelvic infection. Serum lactate level and Gustilo–Anderson grade III injury showed possible associations, although statistical precision was limited. PIs were associated with high medical costs. Early wound management, precise antibiotic therapy, and multidisciplinary approaches are necessary to treat PIs.

## 1. Introduction

The mortality rate among patients with open pelvic fractures (OPFs) caused by high-energy trauma has been reported to range from 7% to 66.7% [1,2,3]. Unlike patients with unstable pelvic fractures, who typically die of hemorrhagic shock, patients with OPFs more commonly die of pelvic soft tissue infections and sepsis [4,5,6,7,8]. These patients often develop severe pelvic infections, including extensive soft tissue infections, necrotizing fasciitis, and pelvic sepsis [5,6,9,10,11].

Several studies have identified shock, advanced sepsis, intra-abdominal injury, and a low Glasgow Coma Scale score as risk factors for the development of pelvic infections and related mortality in patients with OPFs [2,7,8,12]. Early fecal diversion, repeated irrigation and debridement, negative pressure wound therapy, external fixation of the pelvis, and early multidisciplinary approaches have been recommended for treating pelvic infections associated with OPFs [6,8,10,11,13,14,15,16,17,18,19]. Despite these treatment strategies, OPFs are associated with a high incidence of complications, prolonged hospitalization, increased utilization of medical resources, and substantial medical costs [3,5,8,10,11].

This study aimed to examine the clinical characteristics and outcomes of pelvic infection in patients with OPFs and analyze the factors associated with increased medical costs. We hypothesized that advanced wound severity and injury increase the risk of pelvic infection and associated medical costs.

## 2. Materials and Methods

### 2.1. Compliance with Ethical Standards

This study was conducted in accordance with the Declaration of Helsinki and approved by the Institutional Review Board of Ajou University Hospital (AJOUIRB-DB-2023-331; 11 July 2023). The requirement for informed consent was waived for this study due to its retrospective design.

### 2.2. Patients

Patients with OPFs treated at our hospital between March 2016 and February 2023 were reviewed. Pediatric patients (aged <15 years) were excluded from the study. Data collected included patient demographics, associated injuries, surgical procedures, comorbidities, resuscitation methods, injury severity, and radiological and laboratory findings. Sex was recorded as a biological variable (female or male) based on medical records at admission.

Pelvic infection was defined as a broad soft-tissue infection of the pelvic perineum or infection of the deep pelvic organ space. Superficial surgical site infections were not classified as pelvic infections. If the same pathogen was identified in the pelvic wound and bloodstream of a patient diagnosed with sepsis, the condition was defined as pelvic sepsis. Pelvic infections were identified during the index hospitalization without a predefined post-discharge surveillance window; therefore, the reported rates should be interpreted as in-hospital events rather than standardized incidence measures.

Two orthopedic surgeons with more than 10 years of clinical experience analyzed the surgical records and radiological and clinical images. They independently evaluated the Arbeitsgemeinschaft für Osteosynthesefragen/Orthopedic Surgeon Orthopedic Trauma Association (AO/OTA), Young–Burgess, Gustilo–Anderson, and Jones–Powell classifications and the occurrence of pelvic infection. Interobserver agreement was assessed using Cohen’s κ statistics or weighted κ statistics for ordinal variables. Disagreements between reviewers were resolved through consensus discussion based on operative records, radiologic findings, and clinical course review.

The hospitalization period, intensive care unit (ICU) stay period, duration of mechanical ventilator, complications, in-hospital mortality, and medical costs were analyzed.

### 2.3. Statistical Analyses

Normality was assessed using the Kolmogorov–Smirnov test. Continuous variables were compared between the pelvic infection and non-pelvic infection groups using the Mann–Whitney U test or Student’s *t*-test, and the data were presented as medians and interquartile ranges. Categorical variations were analyzed using the chi-squared test or Fisher’s exact test. Logistic regression analysis was used to identify factors associated with pelvic infection. Results were expressed as odds ratios with 95% confidence intervals (CIs). Given the relatively high incidence of pelvic infection, additional analyses were performed to estimate risk ratios using a Poisson regression model with robust variance, which provides a more appropriate measure of association when the outcome is common. Variables with a *p*-value < 0.20 in univariate analysis were considered candidates for inclusion in the multivariable logistic regression model. Missing data were handled using complete-case analysis. The proportion of missing data for each variable was assessed, and variables with substantial missingness (>20%) were not included in the multivariable analysis. Given the small sample size and limited number of events, multiple imputation was not performed to avoid introducing additional model instability. A backward stepwise selection method was applied to identify independent predictors. Given the limited number of events, the number of variables included in the final model was restricted to avoid overfitting, and variables were selected based on both statistical significance and clinical relevance. A causal framework was prespecified to guide variable selection for multivariable analysis. Variables were classified as potential confounders or mediators based on clinical plausibility and prior literature. In particular, anorectal injury and injury severity were considered upstream exposures, whereas surgical interventions such as fecal diversion and laparotomy were considered potential mediators in the causal pathway to pelvic infection. Therefore, to avoid bias from adjustment for intermediates, these variables were not included in the final multivariable model. Multicollinearity among variables included in the regression analyses was assessed using variance inflation factors (VIFs) and condition indices. Model discrimination was assessed using the area under the receiver operating characteristic curve (AUC). All analyses were performed using SPSS (version 23.0; IBM Corp., Armonk, NY, USA) and the R (R Foundation for Statistical Computing, Vienna, Austria) packages GGally (version 2.1.2) and moonBook (version 0.3.1). A two-sided *p* value of <0.05 was considered statistically significant.

## 3. Results

**Figure 1:**
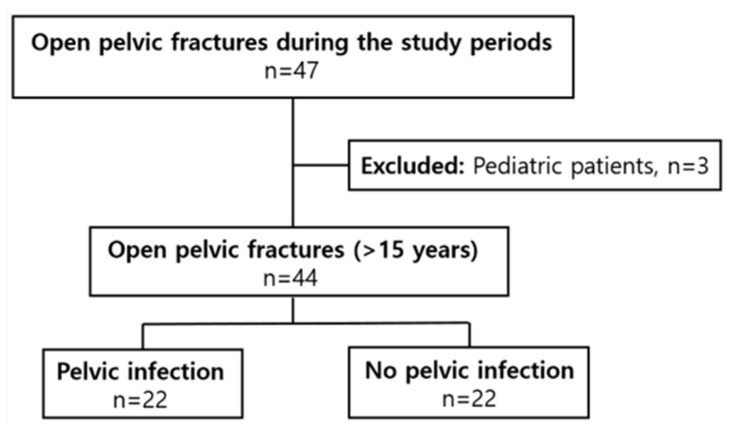
Flow diagram of the study.

Between March 2016 and February 2023, 20,099 patients visited our hospital. Among them, 7249 had an Injury Severity Score (ISS) of ≥16. Of these, 675 had an Abbreviated Injury Scale (AIS) score of ≥4 points, and 47 had OPFs. After excluding three pediatric patients, 44 patients were included in the study (Figure 1). During treatment, 22 patients were identified as having pelvic infections. Interobserver agreement was excellent across all classification systems. Cohen’s κ for the AO/OTA classification was 1.000, while weighted κ values were 1.000 for the Young–Burgess classification, 0.941 for the Gustilo–Anderson classification, and 1.000 for the Jones–Powell classification, indicating almost perfect agreement.

### 3.1. Baseline Characteristics

Baseline characteristics are presented in Table 1. Of the 44 patients, 16 (36.4%) were female and 28 (63.6%) were male. There was no statistically significant difference in sex distribution between the pelvic and non-pelvic infection groups (*p* = 0.347). Patients in the pelvic infection group were older (55.5 [43–64] vs. 37.5 [22–56] years, *p* = 0.060) and had a higher ISS (41.5 [30–43] vs. 26.5 [22–43] years, *p* = 0.051). The pelvic infection group had higher blood lactate levels (8.02 [5.37–10.20] vs. 4.83 [2.93–7.90] mmol/L, *p* = 0.019). However, there were no statistically significant differences between the groups in vital signs or blood gas analysis. The injury mechanism and associated injury rate of AIS ≥ 3 did not differ between the groups. Missing data were minimal, with less than 20% missingness for all variables included in the primary analyses.

### 3.2. Analysis of Transfusion, Resuscitation, and Fecal Diversion

There were no statistically significant differences between the groups in the time required to start the transfusion or the amount of transfusion (Table 2). Laparotomy was performed more frequently in the pelvic infection group [n = 14 vs. 7, *p* = 0.070]. There were no statistically significant intergroup differences in the frequency of resuscitative endovascular balloon occlusion of the aorta, preperitoneal pelvic packing, angioembolization, external fixation, open reduction, or internal fixation. Repeat pelvic packing was performed only in the pelvic infection group (n = 5/10 vs. 0/9, *p* = 0.069). The rate of fecal diversion was higher in the pelvic infection group (n = 15 vs. 3, *p* = 0.001). There was no statistically significant difference between the groups in the time taken until fecal diversion or the percentage of fecal diversions performed within 48 h after arrival at the hospital.

### 3.3. Analysis of Pelvic Fracture Classification and Anorectal Injury

Eighteen patients in the pelvic infection group had Gustilo–Anderson grade III, which was more than twice as high as that in the non-pelvic infection group (n = 8) (*p* = 0.008) (Table 3). There were no differences between the groups in the AO/OTA classification or the Young–Burgess and Jones–Powell classes. Anorectal injury was more common in the pelvic infection group (n = 15 vs. 3, *p* = 0.001).

### 3.4. Logistic Regression for Pelvic Infection

In univariate logistic regression analysis, age, lactate level, ISS, presence of anorectal injury, need for laparotomy, and fecal diversion were statistically significant factors for pelvic infection. Although ISS, laparotomy, and fecal diversion were associated with pelvic infection in univariate analysis, laparotomy and fecal diversion were not included in the multivariable model because they were considered potential mediators rather than confounders; therefore, in the multivariable logistic regression analysis, age (odds ratio 1.02 [95% confidence interval 1.020–1.148], *p* = 0.009) and anorectal injury (36.468 [3.107–427.991], *p* = 0.004) were associated with pelvic infection, whereas serum lactate level (1.319 [0.992–1.755], *p* = 0.018) and Gustilo–Anderson grade III (7.467 [0.987–56.517], *p* = 0.052) showed suggestive but statistically imprecise associations (Table 4). In additional analyses using Poisson regression with robust variance estimation, age (RR 1.023, 95% CI 1.009–1.037), serum lactate level (RR 1.091, 95% CI 1.024–1.163), Gustilo–Anderson grade III injury (RR 2.253, 95% CI 1.089–4.662), and anorectal injury (RR 2.444, 95% CI 1.446–4.129) remained associated with pelvic infection (Supplementary Table S1). No significant multicollinearity was observed. The VIF values ranged from 1.10 to 2.45, and the maximum condition index was 3.33. The model demonstrated acceptable calibration, as indicated by the Hosmer–Lemeshow goodness-of-fit test (*p* = 0.342). The Nagelkerke R^2^ was 0.677, suggesting a moderate-to-substantial proportion of explained variance. Model discrimination was excellent, with an area under the receiver operating characteristic curve (AUC) of 0.926 (95% CI, 0.838–0.983).

### 3.5. Analysis of Complications and Clinical Outcomes

**Figure 2:**
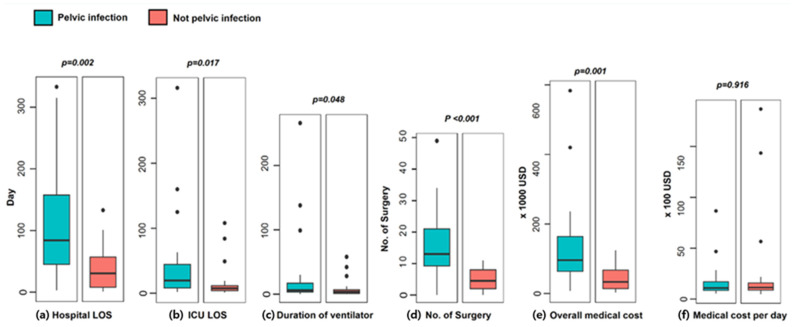
Clinical outcomes and medical costs of open pelvic fractures.

Sepsis, pneumonia, and acute kidney injury occurred more frequently in the pelvic infection group; however, the differences were not significant (Table 5). The pelvic infection group required approximately three times more surgeries during hospitalization than the non-pelvic infection group (13.0 [9–12] vs. 4.5 [2–8], *p* < 0.001) (Figure 2). The hospital length of stay (LOS, 84.0 vs. 30.5 days, *p* = 0.002), ICU stay (19.5 vs. 7.5 days, *p* = 0.017), and duration of mechanical ventilation (6.0 vs. 3.5 days, *p* = 0.048) were more than twice as long in the pelvic infection group as in the non-pelvic infection group. In-hospital mortality was lower in the pelvic infection group; however, the difference was not statistically significant (n = 3 vs. 4, *p* = 1.000). Deaths due to bleeding were encountered only in the non-pelvic infection group. Cases of death due to sepsis or multiorgan dysfunction syndrome (MODS) occurred only in the pelvic infection group.

The overall medical cost was three times higher in the pelvic infection group than in the non-pelvic infection group (95,812 vs. 33,224 USD, *p* = 0.001); however, there was no significant difference in the medical cost per day (1089 vs. 1142 USD, *p* = 1.000) (Figure 2).

The hospital LOS (median [interquartile range]) and number of surgeries for patients with OPFs were 55.5 (15–100) days and 9.0 (3–13), respectively.

### 3.6. Analysis of Microflora in Pelvic Infection of Open Pelvic Fractures

*Enterococcus faecalis* (n = 15) and coagulase-negative *Staphylococcus* (n = 13) were the most common microflora in the pelvic wounds of patients diagnosed with pelvic infections, followed by *Escherichia coli* (n = 11) (Table 6). *E. coli* and *Staphylococcus aureus* were the most common pathogens in the bloodstream (n = 4), followed by *E. faecalis* and *Bacteroides* (n = 3). Eight types of microflora were identified as the cause of pelvic sepsis, with *E. coli* being the most common pathogen (n = 4), followed by *E. faecalis* (n = 3).

## 4. Discussion

This study identified age, serum lactate level, Gustilo–Anderson grade III injury, and anorectal injury as independent risk factors for pelvic infection in patients with OPFs. Furthermore, pelvic infection was associated with high medical costs and resource utilization.

OPFs are life-threatening injuries with high morbidity and mortality [2,5,12,20,21]. Despite advances in damage-control techniques and radiological interventions, OPFs continue to require substantial medical resources [18]. Unlike pelvic fractures that often result in mortality from hemorrhagic shock in the early stages of trauma, OPFs are characterized by delayed morbidity and mortality [2,22]. Frane et al. [23] analyzed open pelvic ring fractures in 19,834 patients and reported that pneumonia, deep organ space surgical site infections, and sepsis were major complications. Dente et al. reported that 45% (n = 9) of the overall mortality in patients with OPFs was associated with late mortality [2]. In this study, pelvic infection was diagnosed in 22 patients with OPFs, and three patients with pelvic infection died of pelvic sepsis. In contrast, death due to hemorrhagic shock occurred only in two patients in the non-pelvic infection group, which was fewer than the deaths caused by sepsis or MODS.

Age is a major prognostic factor in trauma [24]. As the age of a patient increases, the development of comorbidities also increases [25]. Therefore, the Trauma and Injury Severity Score (TRISS), which is a combined scoring system that predicts the survival of trauma patients, adopts age as a major variable [26]. Frane et al. analyzed OPFs based on data from the National Trauma Data Bank and found that patient age was an independent risk factor for OPF complications [23]. A study by Tsugawa et al. reported that 37% of patients with OPFs aged ≥60 years with rectal injury died [14]; Tsugawa et al. suggested that fecal diversion should be considered as a treatment option for older patients with low physiological reserves. Dong et al. reported that among patients with OPFs, the overall mortality was higher in those aged >30 years [27]. Similarly to previous studies, age was found to be an independent risk factor for pelvic infections in our study.

The Faringer zone and Jones–Powell, Gustilo–Anderson, AO/OTA, and Young–Burgess classes were used to evaluate the severity of OPFs and predict their prognosis [2,5,8,20,27,28]. Dente et al. reported the vertical shearing type of Young–Burgess class, Faringer zone I or II, and Gustilo–Anderson grade III as predictive risk factors for overall mortality in OPFs [2]. However, the Young–Burgess class was excluded as a risk factor for late mortality, and Faringer zone I or II and Gustilo–Anderson grade III remained. Dong et al. reported the Gustilo–Anderson grade as a risk factor for late mortality [27], and Cannada et al. reported Jones–Powell class 3 and rectal injury as risk factors predicting mortality in OPFs [5]. Contrary to previous studies, our study did not identify an evaluation tool that could predict overall or late mortality; however, anorectal injury was consistently associated with pelvic infection, whereas serum lactate level and Gustilo–Anderson grade III demonstrated a possible association with substantial statistical uncertainty.

This study showed a relatively higher ISS and a lower revised trauma score than those reported in previous studies; however, the mortality rate was not high. This may reflect the implementation of an updated trauma management strategy, aggressive resuscitation, early wound management, and an early multidisciplinary approach for pelvic infection during the study period. Furthermore, Fitzgerald et al. [29] and Siada et al. [30] reported improved outcomes for OPFs following advances in damage-control surgery and the introduction of current management algorithms.

OPFs often result in high medical costs due to long-term hospitalization and repeated surgeries [3,23]. Chien et al. showed that the average hospitalization period and medical costs for patients with pelvic fracture were 9.3 days and 1475 USD, respectively [31]. Frane et al. [23] reported that the group with complications among patients with OPFs had 2.5 times longer hospitalization periods than the group without complications (17.2 vs. 6.9 days), and Fitzgerald et al. reported that patients with OPFs needed 2.3 times more surgeries than those with closed pelvic fractures [29]. Guo et al. analyzed 46 cases of OPFs and reported a mean hospital LOS of 53.0 ± 37.6 days and mean overall medical cost of 57,049 ± 41,975 USD [3]. The hospital LOS, overall medical cost, and number of surgeries in the pelvic infection group were 2.8 times (84.0 days vs. 30.5 days), 2.9 times (95,812 vs. 33,224 USD), and 2.9 times (13.0 vs. 4.5) higher than those in the non-pelvic infection group, respectively. Therefore, when establishing a treatment strategy for patients with OPFs, efforts to prevent the occurrence of pelvic infections are necessary to save on medical resources and costs.

OPFs are frequently exposed to various microorganisms [10]. Tsugawa et al. reported that *E. coli* and *Bacteroides* are the main pathogens causing infectious complications in OPFs [14]. Salášek et al., who reported surgical site infections in patients with pelvic ring injuries, found that coagulase-negative *Staphylococcus*, *E. faecalis*, *Pseudomonas aeruginosa*, and *E. coli* were the major pathogens [19]. In this study, the frequency of identification of major wound pathogens in pelvic infections was in the following order: *E. faecalis*, coagulase-negative *Staphylococcus*, *E. coli*, and *S. aureus*. However, the types of pathogens detected in the bloodstream of patients with sepsis differed. *E. coli* and *S. aureus* were the most commonly identified bacteria, followed by *E. faecalis* and *Bacteroides*. Coagulase-negative *Staphylococcus* was not cultured from patients with pelvic sepsis. The average number of pathogens identified by culture in the patients with pelvic infections was 5.2. Therefore, patients with OPFs and an increased frequency of mortality or morbidity due to pelvic sepsis or MODS should be administered broad-spectrum antibiotics at the earliest [12]. Furthermore, given the variability of bloodstream pathogens according to our findings, it is crucial to treat patients with severe pelvic infections with sepsis or MODS in consideration of changes in microorganisms.

The interpretation of our multivariable analysis should be considered within a causal framework. Variables such as anorectal injury and overall injury severity represent upstream factors, whereas surgical interventions including fecal diversion and laparotomy are likely to occur downstream in the clinical course and may lie on the causal pathway to pelvic infection. Adjusting for such intermediate variables may introduce bias, including collider stratification bias, and potentially attenuate or distort the true effects of upstream exposures. Therefore, these variables were not included in the final model.

This study has some limitations. First, selection bias may have occurred because this study retrospectively analyzed OPFs that occurred at a single center. However, the single-center design also ensures consistency in trauma management and decision-making, thereby reducing treatment heterogeneity. Second, the number of cases was small because the frequency of OPF is low. During the 7-year study period at this high-volume trauma center, approximately seven patients were diagnosed with OPFs annually. Therefore, a multicenter, large-scale study is required to generalize the results of this study. But despite the limited sample size, several variables demonstrated relatively large effect sizes and consistent associations with pelvic infection. Third, patients in this study had more severe and frequent pelvic infections than those in previous studies. A recent study reported selective fecal diversion in the management of OPFs [14]. However, fecal diversion was performed in most patients with anorectal injuries at our center. Therefore, selection bias may have occurred in relation to the treatment algorithm. Fourth, more detailed standardized criteria for pelvic infection were not applied, which may introduce some degree of diagnostic bias. However, in the context of open pelvic fractures, the distinction between superficial and deep infections is often clinically challenging due to complex anatomy, extensive soft tissue injury, and dynamic progression of infection. To minimize misclassification, infection status was independently assessed by two experienced orthopedic trauma surgeons based on comprehensive clinical, radiological, and operative findings. Fifth, the definition of pelvic sepsis in this study was based on microbiological concordance between pelvic wound and bloodstream isolates, which is a restrictive criterion. This approach likely underestimates the true incidence of pelvic sepsis, as a substantial proportion of clinically relevant infections may be culture-negative due to prior empirical antibiotic use, fastidious or anaerobic organisms, or limitations in culture techniques. Furthermore, this definition does not align with Sepsis-3 criteria, which are based on organ dysfunction rather than microbiological confirmation. Therefore, our reported incidence of pelvic sepsis should be interpreted as a conservative estimate reflecting microbiologically confirmed cases. Future studies should incorporate clinical criteria, including organ dysfunction and radiologic findings, to better capture the full spectrum of pelvic sepsis. Sixth, although sex distribution was reported and did not differ significantly between groups, sex-based subgroup analyses were not performed due to the small cohort size and restricted statistical power. Biological sex may influence trauma response, immune function, and infection risk. Therefore, future studies with larger populations should include adequately powered sex-disaggregated analyses. Seventh, because pelvic infection occurred in 50% of the study population, odds ratios may have overestimated the strength of association relative to risk ratios. Additional Poisson regression analyses with robust variance demonstrated attenuated but directionally consistent effect estimates. Eighth, there is the potential for model overfitting in the multivariable logistic regression analysis. With only 22 events (pelvic infection cases) and four variables included in the final model, the events-per-variable ratio was approximately 5.5, which is below the commonly recommended threshold of 10. This may result in unstable effect estimates and overly wide confidence intervals. Although the model demonstrated excellent discrimination (AUC 0.926, 95% CI 0.838–0.983), the relatively small sample size and limited number of outcome events may increase the risk of model optimism and overfitting. Therefore, the model should be interpreted as exploratory and hypothesis-generating rather than as a validated clinical prediction tool. Indeed, several variables demonstrated wide confidence intervals, reflecting substantial uncertainty in the estimated effects. Therefore, the results of the multivariable analysis should be interpreted with caution and regarded as exploratory and hypothesis-generating rather than confirmatory. This study has additional limitations related to missing data. As a retrospective analysis over a 7-year period, incomplete documentation of clinical variables was unavoidable. We used a complete-case analysis approach, which may introduce bias if the data were not missing completely at random. In particular, missingness related to injury severity or clinical status could potentially affect the observed associations. Therefore, the results should be interpreted with caution.

## 5. Conclusions

Age and anorectal injury were significantly associated with pelvic infection. Serum lactate level and Gustilo–Anderson grade III injury showed possible associations, although statistical precision was limited. Pelvic infections were associated with high medical costs and resource utilization. Aggressive resuscitation, early wound management, precise antibiotic therapy, and early multidisciplinary approaches are necessary for treating pelvic infections and preventing sepsis.

## Figures and Tables

**Table 1:** Baseline demographics.

	No Pelvic Infection	Pelvic Infection	*p* Value
	(n = 22)	(n = 22)	
Age (years), median [IQR]	37.5 [22.0–56.0]	55.5 [43.0–64.0]	0.060
Sex, n			0.347
Female	10	6	
Male	12	16	
Presence of diabetes	2	4	0.660
Mechanism of injury			0.374
MVA	2	3	
MCA	1	3	
Pedestrian	5	8	
Fall from height	11	4	
Struck	2	3	
Penetrating	1	1	
Blunt vs. Penetrating			1.000
Blunt	21	21	
Penetrating	1	1	
Time to hospital (h)	0.9 [0.6–2.4]	0.9 [0.6–1.4]	0.832
Admission			1.000
Transfer	8	7	
Direct	14	15	
ISS	26.5 [22.0–43.0]	41.5 [30.0–43.0]	0.051
RTS	7.329 [5.967–7.841]	6.963 [5.967–7.550]	0.479
TRISS	0.925 [0.680–0.964]	0.827 [0.505–0.936]	0.185
AIS ≥ 3			
Head and neck	4	0	0.116
Thorax	6	11	0.216
Abdomen	4	10	0.106
Extremity	21	21	1.000
Vital signs and gas analysis on arrival			
SBP (mmHg)	120.0 [86.0–137.0]	97.5 [74.0–121.0]	0.133
DBP (mmHg)	78.5 [65.0–100.0]	67.0 [47.0–87.0]	0.213
PR (min^−1^)	98.5 [86.0–114.0]	107.0 [91.0–131.0]	0.240
RR (min^−1^)	20.5 [18.0–25.0]	24.0 [19.0–30.0]	0.213
GCS	14.0 [6.0–15.0]	14.0 [12.0–15.0]	0.748
Lactate (mmol/L)	4.83 [2.93–7.90]	8.02 [5.37–10.20]	0.019
Base excess (mmol/L)	−7.90 [−11.00 to −3.10]	−9.75 [−13.20 to −7.10]	0.173

Abbreviations: AIS, Abbreviated Injury Scale; DBP, diastolic blood pressure; GCS, Glasgow Coma Scale; IQR, interquartile range; ISS, Injury Severity Score; MCA, motorcycle accident; MVA, motor vehicle accident; PR, pulse rate; RR, respiratory rate; RTS, revised trauma score; SBP, systolic blood pressure; TRISS, Trauma and Injury Severity Score.

**Table 2:** Pelvic fracture classification and anorectal injury.

	No Pelvic Infection	Pelvic Infection	*p* Value
	(n = 22)	(n = 22)	
Gustilo–Anderson grade			0.008
1	2	1	
2	12	3	
3	8	18	
AO/OTA class			0.578
A	7	6	
B	4	7	
C	11	9	
Young–Burgess class			0.698
APC	7	11	
LC	3	2	
VS	5	6	
Jones–Powell class			0.212
1	5	1	
2	6	7	
3	11	14	
Anorectal injury	2	13	0.001

Abbreviations: AO/OTA, Arbeitsgemeinschaft für Osteosynthesefragen/Orthopedic Surgeon’s Orthopedic Trauma Association; APC, anteroposterior compression; LC, lateral compression; VS, vertical shearing.

**Table 3:** Transfusion, resuscitation, and fecal diversion.

	No Pelvic Infection	Pelvic Infection	*p* Value
	(n = 22)	(n = 22)	
Transfusion			
Time to transfusion (min^−1^)	16.5 [9.0–43.5]	13.0 [7.0–20.0]	0.231
RBC for first 24 h	18.0 [6.0–27.5]	19.5 [9.0–33.0]	0.929
FFP for first 24 h	17.5 [6.0–28.0]	19.0 [5.0–33.0]	0.824
PLT for first 24 h	4.5 [0.5–9.5]	7.5 [2.0–16.0]	0.355
Massive transfusion	11	16	0.216
Resuscitation and surgical procedures			
REBOA	5	3	0.696
PPP	9	10	1.000
Repeated packing	0	5	0.069
Angioembolization	8	9	1.000
Laparotomy	7	14	0.070
External fixation	4	5	1.000
ORIF	7	9	0.754
Fecal diversion	3	15	0.001
Time to fecal diversion (h)	48.0 [38.0–71.0]	46.0 [6.0–118.0]	0.800
Fecal diversion < 48 h	2	8	1.000

Abbreviations: FFP, fresh frozen plasma; ORIF, open reduction and internal fixation; PPP, preperitoneal pelvic packing; PLT, platelet; RBC, red blood cell; REBOA, resuscitative endovascular balloon occlusion of the aorta.

**Table 4:** Univariate and multivariate logistic regression for pelvic infection.

	Univariate Analysis			Multivariate Analysis		
	Odds Ratio (95% CI)		*p* Value	Odds Ratio (95% CI)		*p* Value
Age	1.031	(0.997–1.066)	0.077	1.082	(1.020–1.148)	0.009
Serum lactate	1.183	(0.994–1.408)	0.058	1.319	(0.992–1.755)	0.018
Anorectal injury	14.444	(2.682–77.796)	0.002	36.468	(3.107–427.991)	0.004
Gustilo–Anderson grade III	7.875	(1.964–31.547)	0.004	7.467	(0.987–56.517)	0.052
ISS	1.047	(0.995–1.101)	0.077			
Laparotomy	3.750	(1.076–13.073)	0.038			
Fecal diversion	13.571	(2.991–61.586)	0.001			

Nagelkerke’s R^2^ = 0.677; Hosmer–Lemeshow’s goodness-of-fit test, *p* = 0.342, 84.1%; Abbreviations: CI, confidence interval; ISS, injury severity score.

**Table 5:** Complications and in-hospital mortality.

	No Pelvic Infection	Pelvic Infection	*p* Value
	(n = 22)	(n = 22)	
Complications			
DVT	3	2	1.000
PTE	1	0	1.000
Sepsis	1	6	0.099
Pneumonia	2	4	0.660
AKI.ARF	1	5	0.188
In-hospital mortality	4	3	1.000
Cause of death			0.072
Bleeding	2	0	
Sepsis/MODS	0	3	
Brain	1	0	
Others	1	0	
Time to death (h)	57.5 [28.5–110.5]	63.0 [54.5–222.5]	0.857

Abbreviations: AKI, acute kidney injury; ARF, acute renal failure; DVT, deep vein thrombosis; MODS, multiorgan dysfunction syndrome; PTE, pulmonary thromboembolism.

**Table 6:** Pathogen analysis of patients with pelvic infection.

Pathogen/Location	Pelvic Wound, n	Blood Stream, n	Pelvic Sepsis, n
*Enterococcus faecalis*	15	3	3
Coagulase-negative *Staphylococcus*	13	0	0
*Escherichia coli*	11	4	4
*Staphylococcus aureus*	8	4	2
*Pseudomonas aeruginosa*	8	1	0
*Acinetobacter baumannii*	8	1	1
*Enterococcus faecium*	7	2	1
*Enterococcus avium*	7	0	0
*Corynebacterium striatum*	5	0	0
*Klebsiella pneumoniae*	5	2	2
*Candida*	4	2	2
*Stenotrophomonas maltophilia*	2	0	0
*Enterococcus hirae*	1	0	0
*Enterobacter cloacae*	1	1	0
*Bacillus* species	1	0	0
*Streptococcus agalactiae*	1	1	1
*Citrobacter braakii*	1	0	0
*Proteus mirabi*	1	0	0
*Staphylococcus epidermidis*	0	2	0
*Streptococcus mitis* group	0	1	0
*Bacteroides*	0	3	0
*Haemophilus influenzae*	0	1	0
*Staphylococcus lugdunensis*	0	1	0
*Clostridium* species	0	1	0

## Data Availability

The data supporting the conclusions of this article will be made available by the authors on request because the data may contain patients’ personal information.

## Supplementary Materials

The following supporting information can be downloaded at: https://www.mdpi.com/article/10.3390/healthcare14101328/s1, Supplementary Table S1. Comparison of effect estimates derived from Poisson regression with robust variance (risk ratios).

## Abbreviations

The following abbreviations are used in this manuscript:

OPF	Open pelvic fracture
AO/OTA	Arbeitsgemeinschaft für Osteosynthesefragen/Orthopedic Surgeon Orthopedic Trauma Association
ICU	Intensive care unit
CI	Confidence interval
ISS	Injury Severity Score
AIS	Abbreviated Injury Scale
IQR	Interquartile range
DBP	Diastolic blood pressure
GCS	Glasgow Coma Scale
MCA	Motorcycle accident
MVA	Motor vehicle accident
PR	Pulse rate
RR	Respiratory rate
RTS	Revised trauma score
SBP	Systolic blood pressure
TRISS	Trauma and Injury Severity Score
APC	Anteroposterior compression
LC	Lateral compression
VS	Vertical shearing
FFP	Fresh frozen plasma
ORIF	Open reduction and internal fixation
PPP	Preperitoneal pelvic packing
PLT	Platelet
RBC	Red blood cell
REBOA	Resuscitative endovascular balloon occlusion of the aorta
AKI	Acute kidney injury
ARF	Acute renal failure
LOS	Length of stay
MODS	Multiorgan dysfunction syndrome
DVT	Deep vein thrombosis
PTE	Pulmonary thromboembolism
