# Supplementary material for: Risk Factors, Clinical Outcomes, and Medical Costs of Pelvic Infection After Open Pelvic Fractures: A 7-Year Retrospective Observational Study at a Single Trauma Center
Source: Healthcare (Basel). 2026 May 13;14(10):1328. doi: 10.3390/healthcare14101328 (PMC13205321; doi:10.3390/healthcare14101328)
Supplement: Supplementary file 1 [file healthcare-14-01328-s001.zip › healthcare-4220945-supplementary.pdf]

Supplementary Table S1. Comparison of effect estimates derived from Poisson regression with robust variance (risk ratios).

Variable	Risk Ratio (RR)	95% CI	p value
Age	1.023	1.009–1.037	0.001
Lactate	1.091	1.024–1.163	0.007
Gustilo–Anderson grade III	2.253	1.089–4.662	0.028
Anorectal injury	2.444	1.446–4.129	<0.001
